# Economic burden of mental illness in Pakistan: an estimation for the year 2020 from existing evidence

**DOI:** 10.1192/bji.2023.4

**Published:** 2023-08

**Authors:** Mohsin Hassan Alvi, Tehmina Ashraf, Tayyeba Kiran, Nasir Iqbal, Anil Gumber, Anita Patel, Nusrat Husain

**Affiliations:** 1 Research Fellow, Pakistan Institute of Living and Learning, Karachi, Pakistan. Email: mohsin.h.alvi@pill.org.pk; 2 Research Assistant, Remedial Center Hospital and Nursing Home, Karachi, Pakistan; 3 Assistant Director Research and Development, Pakistan Institute of Living and Learning, Karachi, Pakistan; 4 Associate Professor, Pakistan Institute of Development Economics, Islamabad, Pakistan; 5 Principal Health Economist, College of Health, Wellbeing and Life Sciences, Sheffield Hallam University, Sheffield, UK; 6 Health Economist, Anita Patel Health Economics Consulting, London, UK; 7 Professor of Psychiatry, Division of Psychology & Mental Health, University of Manchester, Manchester, UK

**Keywords:** Pakistan, economic burden, mental illness, extrapolation, cost

## Abstract

This report is based on the extrapolation to 2020 of data on the economic burden of mental illnesses in Pakistan in 2006. Given the resultant estimated high economic burden of mental illness in the country (£2.97 billion in 2020), we advocate a revised budget allocation to mental healthcare. As a resource-scarce nation that is entangled in natural disasters, Pakistan needs cost-effective psychological interventions such as culturally adapted manual-assisted problem-solving training (C-MAP) for the prevention of self-harm and suicide and to move towards attaining the United Nations’ Sustainable Development Goals (SDGs). Although government has taken initiatives to support healthcare services (such as the Sehat Sahulat Program for universal health coverage), there is still a need to implement a cost-effective national digital model for mental healthcare such as the Agha Khan Development Network Digital Health Programme.

Despite 10–16%^1^ of Pakistan's population suffering from mental illnesses and this being a major contributor to the overall burden of disease, mental illness remains overlooked in setting national priorities in this and other low- and middle-income countries (LMICs). LMICs are more vulnerable to natural disasters and, owing to people's poor socioeconomic status, mental health crises emerging from such disasters are also neglected.^2^ According to the INFORM disaster risk index for 2019, Pakistan is ranked 18th out of 191 countries; flooding events are the most common disaster, causing an average annual loss around $1 billion.^3^ The worst monsoon flood in 2022 displaced more than 3.1 million people in Pakistan.^4^ These events are associated with psychological distress due to financial problems and displacement.^5^ Considering the burden of psychological trauma, the International Federation of Red Cross and Red Crescent Societies (IFRC) has introduced a psychoeducational package offering psychological first aid.^4^ Although service provision for mental disorders is considered an integrated part of efficient healthcare systems, most LMICs fail to address the needs of at-risk populations (Fig. 1).^6^ Despite contributing 8.9% of the disease burden, mental illness remains neglected in such settings.^6^ Apart from a few studies conducted over the years, the associated economic cost remains unclear. To fill this gap, we have estimated the burden of mental illness in Pakistan.

**Fig. 1:**
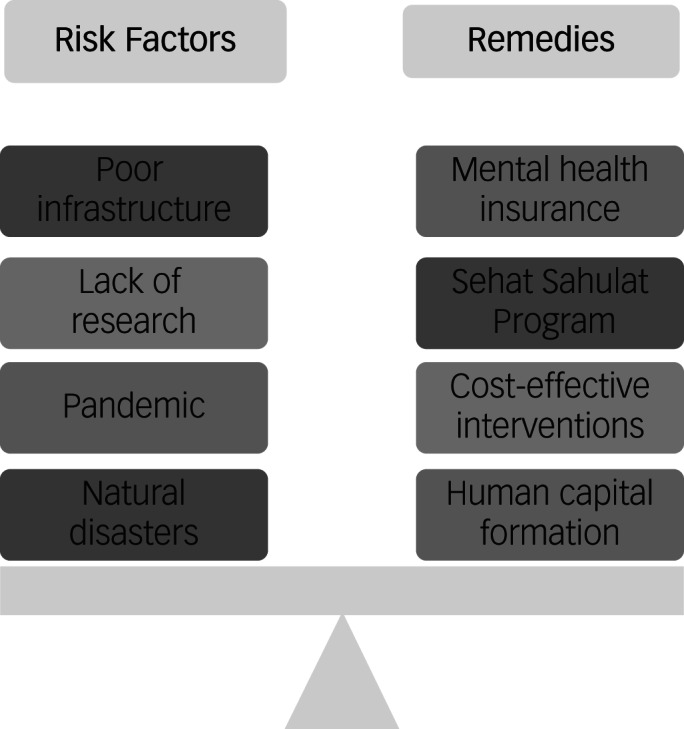
Risk and protective factors of mental health in Pakistan.

## Method and results

We extrapolated the costs of mental illness in Pakistan from 2006 data (as reported in 2016 by Malik & Khan^7^) to 2020 using the overall gross domestic product (GDP) deflator. This extrapolation showed that the economic burden of mental illness is rising at an alarming level, from 250.5 billion Pakistani rupees (PKR) in 2006 to 616.9 billion PKR in 2020 (Table 1). Only a fraction of the total health budget is allocated to mental health. In LMICs such as Pakistan, where the burden of mental illnesses is high, allocating only 2.4 billion PKR (0.4%)^1,8–10^ from a total health budget of 617.2 billion PKR will not help much in overcoming the huge challenge.^10^ Our calculations suggest that the existing budget is only able to cover <2% of the entire economic burden of mental illnesses for the year 2020. A limited budget might not be a big problem if it is utilised effectively, but in LMICs effective allocation is also a challenge. A total of 11% of the country's mental health budget is utilised in hospital psychiatric units; the remaining 89% is for other mental health facilities.^11^ These hospital psychiatric units are mostly located in urban areas, and these are overburdened in most cities. Furthermore, patients are forced to manage the rising cost of psychiatric medication; over 2 million of Pakistan's population are living below the poverty line^12^ and only 5% of the population has access to free medicines.^11^ As Pakistan's public health sector has yet to fully recognise psychology as a profession, the number of psychology and psychiatry professionals in the country is limited: 20% of graduates prefer to settle in foreign countries because of the lack of opportunities in Pakistan.^11^ The fact that Pakistan has no comprehensive mental health policy makes it a neglected subject, not only in terms of poor institutional infrastructure but also in terms of legislation.^13^

**Table 1:** Estimated economic burden of mental illness for the year 2020 in Pakistan^a^

Year	GDP index	Economic burden, billion PKR^b^	Economic burden, billion £	Currency conversion (PKR to £)
2006	100.00	250.5	2.26	(£1 = 110.96 PKR)
2020	346.29	616.9	2.97	(£1 = 207.71 PKR)

GDP, gross domestic product; PKR, Pakistani rupees.

a. The 2020 economic burden data were extrapolated from the 2006 data using the Pakistan GDP deflator.^14^

b. The 2006 economic burden data are from Malik & Khan.^7^

## Ways forward

### Cost-effective psychological interventions

To address the burden of poor mental health, we need robust research on evaluation of treatments with minimum cost and maximum benefits. Depression, suicidal behaviour and other mental health problems are probably rising since COVID-19. Pakistan's journey towards the attainment of the United Nations’ Sustainable Development Goals (SDGs) for 2030 (https://sdgs.un.org/goals) demands effective prevention of mental illnesses and interventions to reduce the mental illness burden. Effective interventions can help by improving health and economic outcomes. Although efforts have already been started in Pakistan, researchers are struggling to present local evidence to the relevant stakeholders on how much should be invested in different healthcare projects. For example, it is estimated that a minimum of US$16 254 is required for reduction of self-harm (a psychosocial intervention delivery cost to gain one unit of output).^15^ Some interventions have been tested and recommended: for example, a culturally adapted manual-assisted problem-solving training (C-MAP) intervention has been found to be cost-effective in reduction of self-harm in Pakistan.^15^ To develop clinically effective and cost-effective interventions that are locally relevant, Pakistan needs to invest more in mental health research and capacity building.

### Universal health coverage

In Pakistan, mental health services are now included in the Sehat Sahulat Program (a health insurance scheme for underprivileged citizens) but to a limited extent. Mental illness is often not covered by insurance, and government spending is considered the major source of financing for affordable healthcare. Pakistan's neighbouring countries, such as India and China, have allocated a separate share of their budget to mental healthcare. Efficient and effective budget allocation plays a vital role in cost minimisation. The mental health budget should be allocated with a particular focus on low-resource districts, to avoid patients and their families having to travel to tertiary care hospitals in better resourced districts of Pakistan. Subsidised mental health services can be provided by including all mental health treatments in the Sehat Sahulat Program or other health coverage programmes. There are some good international examples, such as the Agha Khan Development Network Digital Health, which operates in South-Central Asia; we need to implement a cost-effective digital model for healthcare at a national level.

### Refocusing policy and funding

As children and youth are the future of any nation, they should hold greater focus in Pakistan's mental health policy. Currently, funding is targeted at specialist services only, but low-intensity therapies have also been shown to be cost-effective for treating mental illnesses.^16^ Timely evaluation and updated evidence could be the key to meeting the mental health needs of the country, achieving the United Nations' 2030 SDGs and ensuring progress in Pakistan.

## Data Availability

The data that support the findings of this study are openly available.
